# Comparison of genomic signatures of selection on *Plasmodium falciparum* between different regions of a country with high malaria endemicity

**DOI:** 10.1186/s12864-015-1746-3

**Published:** 2015-07-16

**Authors:** Craig W. Duffy, Samuel A. Assefa, James Abugri, Nicholas Amoako, Seth Owusu-Agyei, Thomas Anyorigiya, Bronwyn MacInnis, Dominic P. Kwiatkowski, David J. Conway, Gordon A. Awandare

**Affiliations:** Pathogen Molecular Biology Department, London School of Hygiene and Tropical Medicine, London, WC1E 7HT UK; West African Centre for Cell Biology of Infectious Pathogens (WACCBIP), Department of Biochemistry, Cell and Molecular Biology, University of Ghana, Box LG 54, Volta Road, Legon, Accra, Ghana; Department of Applied Chemistry and Biochemistry, University for Development Studies, Tamale, Ghana; Kintampo Health Research Centre, Kintampo, Ghana; Navrongo Health Research Centre, Navrongo, Ghana; Wellcome Trust Sanger Institute, Hinxton, UK; Wellcome Trust Centre for Human Genetics, University of Oxford, Oxford, UK

## Abstract

**Background:**

Genome wide sequence analyses of malaria parasites from widely separated areas of the world have identified contrasting population structures and signatures of selection. To compare relatively closely situated but ecologically contrasting regions within an endemic African country, population samples of *Plasmodium falciparum* clinical isolates were collected in Ghana from Kintampo in the central forest-savannah area, and Navrongo in a drier savannah area ~350 km to the north with more seasonally-restricted transmission. Parasite DNA was sequenced and paired-end reads mapped to the *P. falciparum* reference genome.

**Results:**

High coverage genome wide sequence data for 85 different clinical isolates enabled analysis of 121,712 single nucleotide polymorphisms (SNPs). The local populations had similar proportions of mixed genotype infections, similar SNP allele frequency distributions, and eleven chromosomal regions had elevated integrated haplotype scores (|iHS|) in both. A between-population Rsb metric comparing extended haplotype homozygosity indicated a stronger signal within Kintampo for one of these regions (on chromosome 14) and in Navrongo for two of these regions (on chromosomes 10 and 13). At least one gene in each of these identified regions is a potential target of locally varying selection. The candidates include genes involved in parasite development in mosquitoes, members of variant-expressed multigene families, and a leading vaccine-candidate target of immunity.

**Conclusions:**

Against a background of very similar population structure and selection signatures in the *P. falciparum* populations of Ghana, three narrow genomic regions showed evidence indicating local differences in historical timing or intensity of selection. Sampling of closely situated populations across heterogeneous environments has potential to refine the mapping of important loci under temporally or spatially varying selection.

**Electronic supplementary material:**

The online version of this article (doi:10.1186/s12864-015-1746-3) contains supplementary material, which is available to authorized users.

## Background

Malaria is a globally important disease which exhibits major differences in local epidemiology and ecology, with great variation within Africa where most cases are caused by *Plasmodium falciparum* [1]. Local differences in selection by infectious diseases has impacted on human genetic variation [2], and malaria parasites have played a significant role in this [3]. Conversely, selection operating on parasites must vary locally due to varying transmission ecology, differences in innate susceptibility of mosquito or human populations, degrees of acquired immunity in humans, or drug pressure. Malaria parasites in highly endemic regions are subject to competition due to superinfection by different genotypes, and are subject to strong acquired immune responses, whereas low endemicity requires more prolonged maintenance of asexual parasite infection as there are only rare opportunities for transmission of sexual stages during seasons when mosquitoes are present [4]. Malaria parasites have a compact ~23 Megabase haploid genome of 14 chromosomes containing more than 5,000 genes [5, 6], with a high recombination rate (approximately 10–20 kb per centiMorgan for *P. falciparum*) in a brief diploid stage that occurs after parasite mating in the mosquito each transmission cycle [7], and thus should allow relatively efficient mapping of signatures of natural selection [8].

Recent genome-wide analyses of *Plasmodium falciparum* revealed significant global population structure [9, 10], consistent with previous microsatellite genotyping surveys that indicated substantial divergence between Southeast Asia and Africa, and slight differentiation between East and West Africa [11, 12]. Local subdivisions in *P. falciparum* population structure in Southeast Asia may be important for understanding the current emergence of Artemisinin antimalarial drug resistance [10, 13–16]. In contrast, there is little evidence of parasite population subdivision within the large endemic region of West Africa [9, 10, 17, 18], although infection endemicity ranges from very high in the south where there is abundant rainfall to low in the north where rainfall is limited [1]. Initial analyses to scan for loci under selection within West Africa have been performed on populations sampled from Senegal [19, 20], The Gambia [20–22], and Guinea [17]. Differences between populations are evident in signatures of selection surrounding genes involved with chloroquine (*mdr1* and *crt*) and anti-folate (*dhfr* and *dhps*) resistance that reflect differences in historical drug use among the countries [17, 19, 22]. Direct comparison between a highly endemic population in Guinea and a population with lower endemicity in The Gambia indicated another locus with alleles at highly differentiated frequencies, containing the gametocyte development gene *gdv1* that is essential for parasite transmission [17]. However, more local differences between parasite populations within an endemic country in Africa have not yet been investigated by genome-wide surveys with adequate sample sizes to detect differences in selective signatures.

**Fig. 1:**
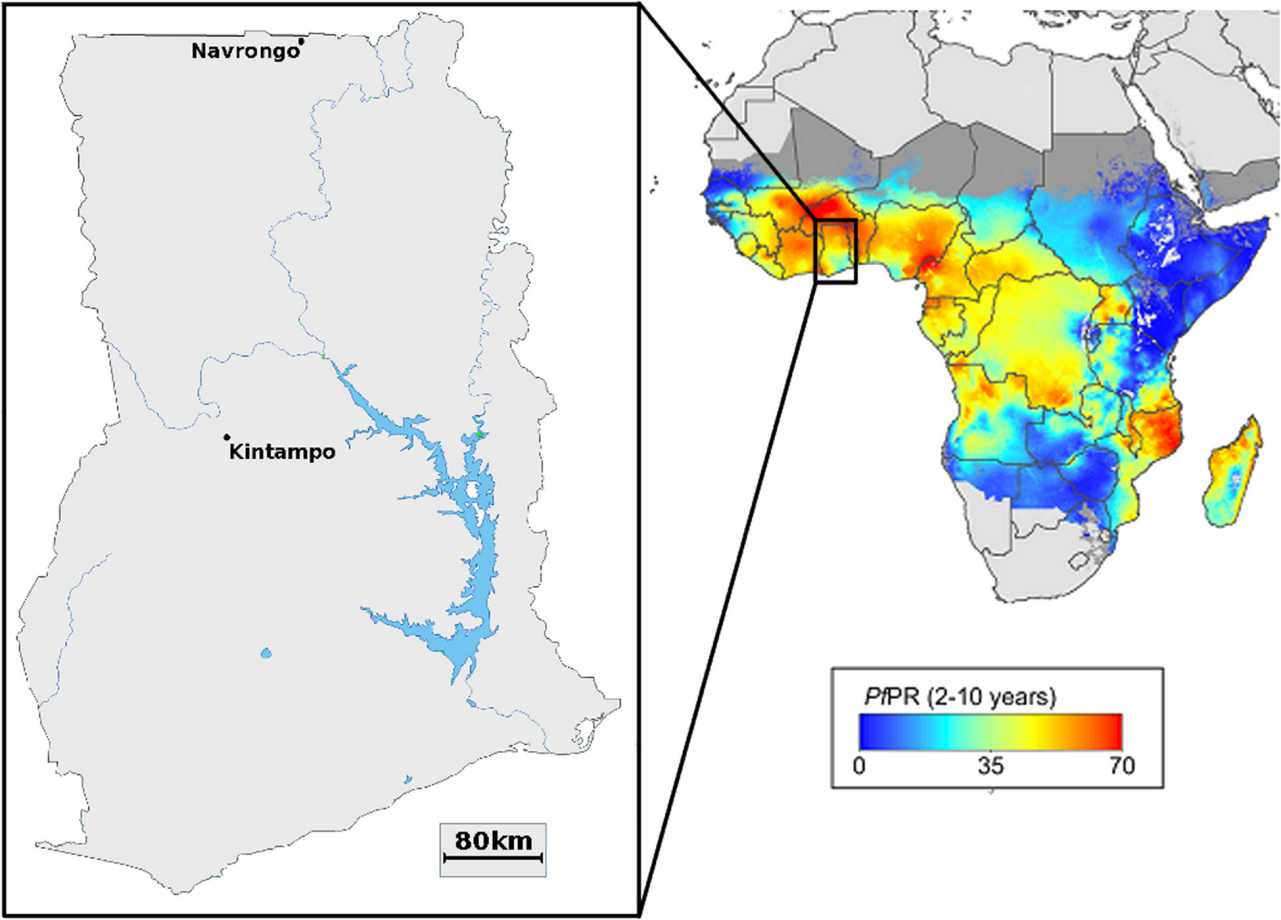
Location of sites in Ghana at which malaria infections were sampled for study of *Plasmodium falciparum* genome sequences. The epidemiology of malaria has been previously described in Kintampo [23, 24] and Navrongo [25, 26], which are separated by approximately 350 km. The Figure incorporates graphics from d-maps.com (http://d-maps.com/carte.php?num_car=26832&lang=en) and the Malaria Atlas Project (*Pf*PR shading refers to the geographical projected estimate of *P. falciparum* slide positive percent prevalence in children aged between 2 and 10 years) [1]

To test for selection that might occur due to varying patterns of transmission seasonality, varying antimalarial drug use, or other causes, *P. falciparum* population samples were analysed from two different areas of Ghana (Fig. 1). Clinical isolates from *P. falciparum* malaria patients at two sites separated by ~350 km were sequenced and the resulting high quality genome sequence data from 85 isolates were analysed. Malaria transmission in Kintampo, in the central Forest-Savannah transitional zone, occurs for much of each year [23, 24], while Navrongo, in the Sudan Savannah zone near the northern border with Burkina Faso, experiences more markedly seasonal transmission [25, 26]. Major mosquito vectors in Ghana include *An. gambiae sensu stricto* (s.s.)(‘S molecular form’), *An. coluzzii* (previously termed as the ‘M molecular form’ of *An. gambiae*), *An. arabiensis*, and *An. funestus* [24, 25, 27], with survey data suggesting that *An. arabiensis* and *An. coluzzii* may be more common in the north than in central areas of the country [27]. The overall genome-wide SNP patterns in the two *P. falciparum* populations analysed here were not significantly different, and most of the strong selective signatures were evident in both populations, several of these signatures mapping to loci encoding known targets of antimalarial drugs and immunity. However, haplotype-based tests indicated a small number of chromosomal regions at which signatures of recent directional selection were stronger in one or other population, and these putatively contain one or more genes under locally varying selection with potential relevance to malaria control.

## Results

### Parasite allele frequency distributions and within-host infection diversity

Illumina short read sequence data obtained from 101 of the clinical infection isolates (Additional file 1: Table S1) were mapped to the 3D7 *P. falciparum* reference genome sequence, enabling high quality genome-wide SNP calling and analysis for 85 isolates (45 from Kintampo and 40 from Navrongo) passing the quality filtering described in the Methods (Additional file 1: Table S1). This identified 121,712 biallelic SNPs in the combined population, with majority read allele calls for all isolates across a total of 107,221 positions, and <5 % missing isolate data for the remaining 14,491 SNPs. For 87,066 SNPs (72 % of the total) the minor frequency allele in the total population sample was observed as the majority read allele for only a single isolate, similar to proportions in previously examined West African populations with approximately similar sample sizes [9, 17, 19, 22], leaving 34,646 non-singleton SNPs in the overall dataset. The majority (65 %) of all SNPs were observed within genes, as a more extreme A + T bias within intergenic regions (87 % A + T, versus 70 % A + T in coding sequences) restricts ability to map reads uniquely [9].

**Fig. 2:**
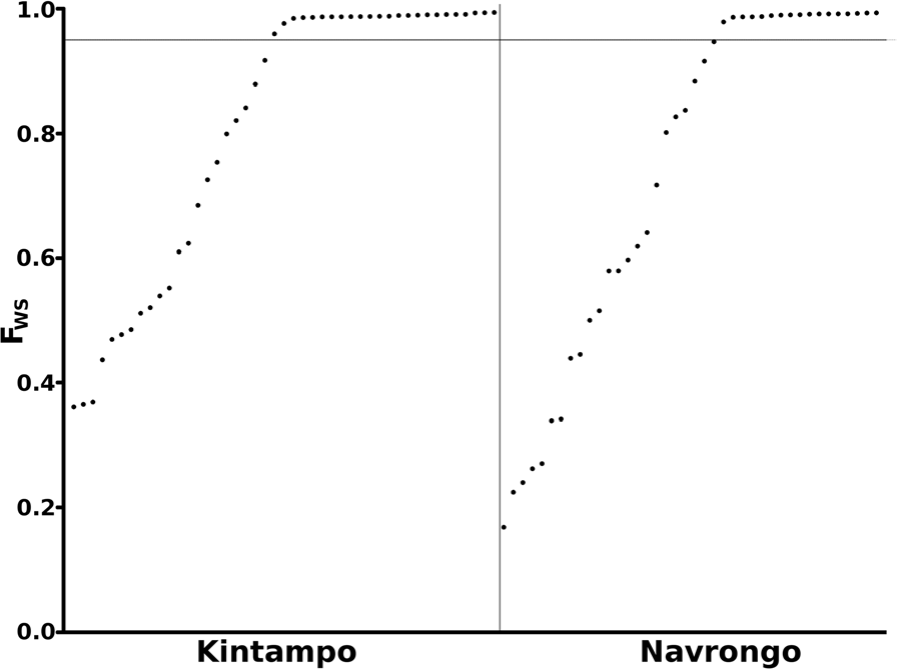
Within-infection *F*_*ws*_ fixation indices for each clinical isolate with genome-wide SNP data from Kintampo (*N* = 45) and Navrongo (*N* = 40), ordered by increasing index value. The distribution of *F*_*ws*_ values was not significantly different between the populations (Mann–Whitney test, *P* = 0.52). The horizontal line marks *F*_*ws*_ = 0.95, above which an isolate may be considered to contain a single predominant genotype; 24 (53 %) of 45 isolates in Kintampo and 20 (50 %) of 40 isolates in Navrongo

The within-infection diversity in each isolate was assessed using the *F*_WS_ fixation index [9, 28]. The distribution of *F*_WS_ scores was similar across isolates in each local population, ranging from 0.36 - 0.99 (mean 0.81) in Kintampo, and from 0.17 - 0.99 (mean 0.74) in Navrongo (Fig. 2, Mann–Whitney *P* = 0.52). The proportions of isolates with *F*_WS_ scores above 0.95, indicating infections dominated by single genotypes at the time of sampling, were 24 (53 %) of 45 isolates in Kintampo and 20 (50 %) of 40 isolates in Navrongo. Within each isolate, the majority allele at each SNP (with the highest number of mapped reads) was counted towards analyses of population-based allele frequencies for the following tests.

**Fig. 3:**
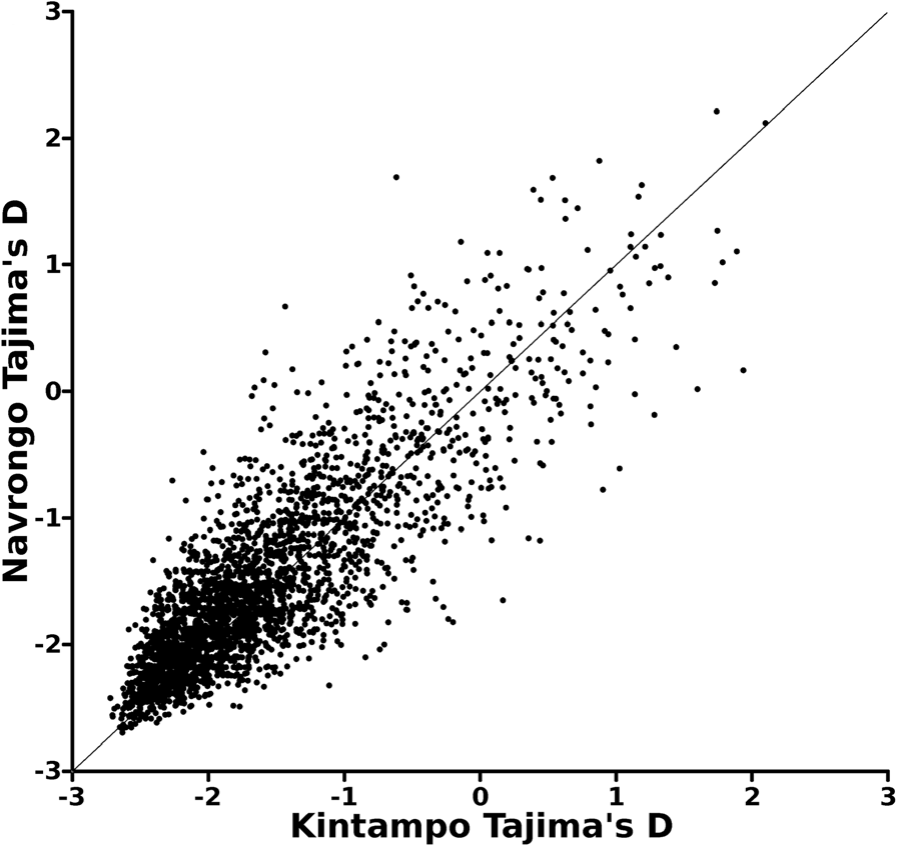
Correlation of Tajima’s D indices shows similar overall genome-wide allele frequency distributions of SNPs within genes in each of the two Ghanaian population samples, Kintampo (*N* = 45) and Navrongo (*N* = 40). The scatterplot compares Tajima’s D indices in both populations for each of 4,048 genes containing 3 or more SNPs (Correlation *R* ^*2*^ = 0.71)

The SNP allele frequency spectra in each of the two local populations were examined by calculation of Tajima’s D value for the 4,048 genes with 3 or more SNPs in at least one of the two populations (Additional file 2: Figure S1 and Additional file 3: Dataset S1). There was a strong correlation between the two populations (*R*^*2*^ = 0.71) in the distributions of Tajima’s D values across all genes (Fig. 3). Tajima’s D values of above 1.0 were observed for 27 genes in Kintampo and 24 genes in Navrongo, with 12 of these genes having values of above 1.0 in both populations (Additional file 3: Dataset S1). Reference genomic map positions and annotations may be viewed on PlasmoDB (www.plasmodb.org) [5] using the gene ID numbers.

### Testing for differentiation between populations

**Fig. 4:**
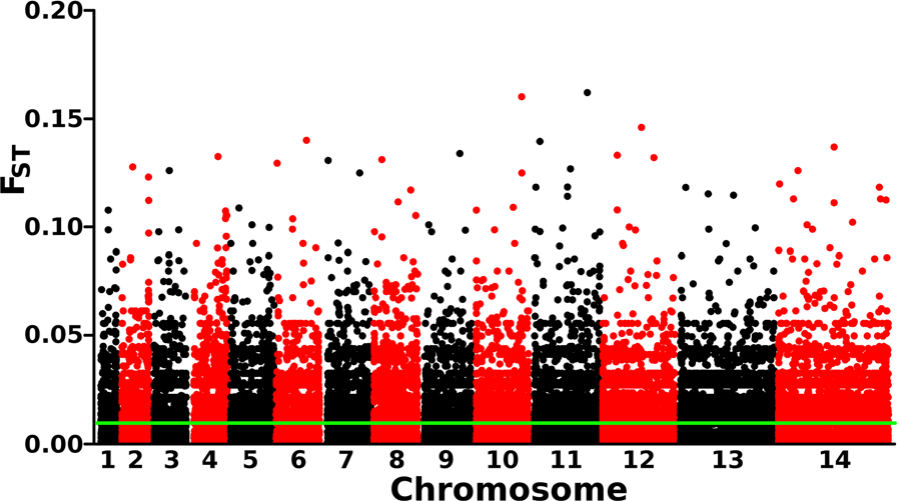
Genome-wide *F*_ST_ scan for differentiation between the Kinpampo (*N* = 45) and Navrongo (*N* = 40) population samples, for each of 34,646 non-singleton SNPs genome-wide. The green line indicates the genome-wide mean *F*_ST_ = 0.012. The positions of SNPs with *F*_ST_ values above 0.1 are shown in Additional file 1: Table S2, but the numbers of these did not differ from null expectations based on sampling from a single pooled population

Principal component analysis using the 107,221 SNPs with no missing data in the complete dataset of 85 isolates did not show any separation between isolates sampled from the Kintampo and Navrongo populations (Additional file 2: Figure S2). To scan for individual SNPs which might have allele frequency differentiation between the populations, the fixation index *F*_ST_ was calculated for each of the 34,646 non-singleton SNPs genome-wide (Fig. 4). There was minimal differentiation between populations (mean *F*_ST_ = 0.012, median = 0.006), and only 51 SNPs had *F*_ST_ values above 0.1, with the highest *F*_ST_ value being 0.16 (these SNPs and their allele frequencies in each of the populations are listed in Additional file 1: Table S2). The observed numbers of SNPs with *F*_ST_ values above 0.1 was not significantly different from null expectations due to sampling variance (equivalent to the 32^nd^ percentile of the distribution derived by random sampling of two population subsets of 45 and 40 isolates from a single combined population sample).

### Evidence of directional selection within local populations

**Fig. 5:**
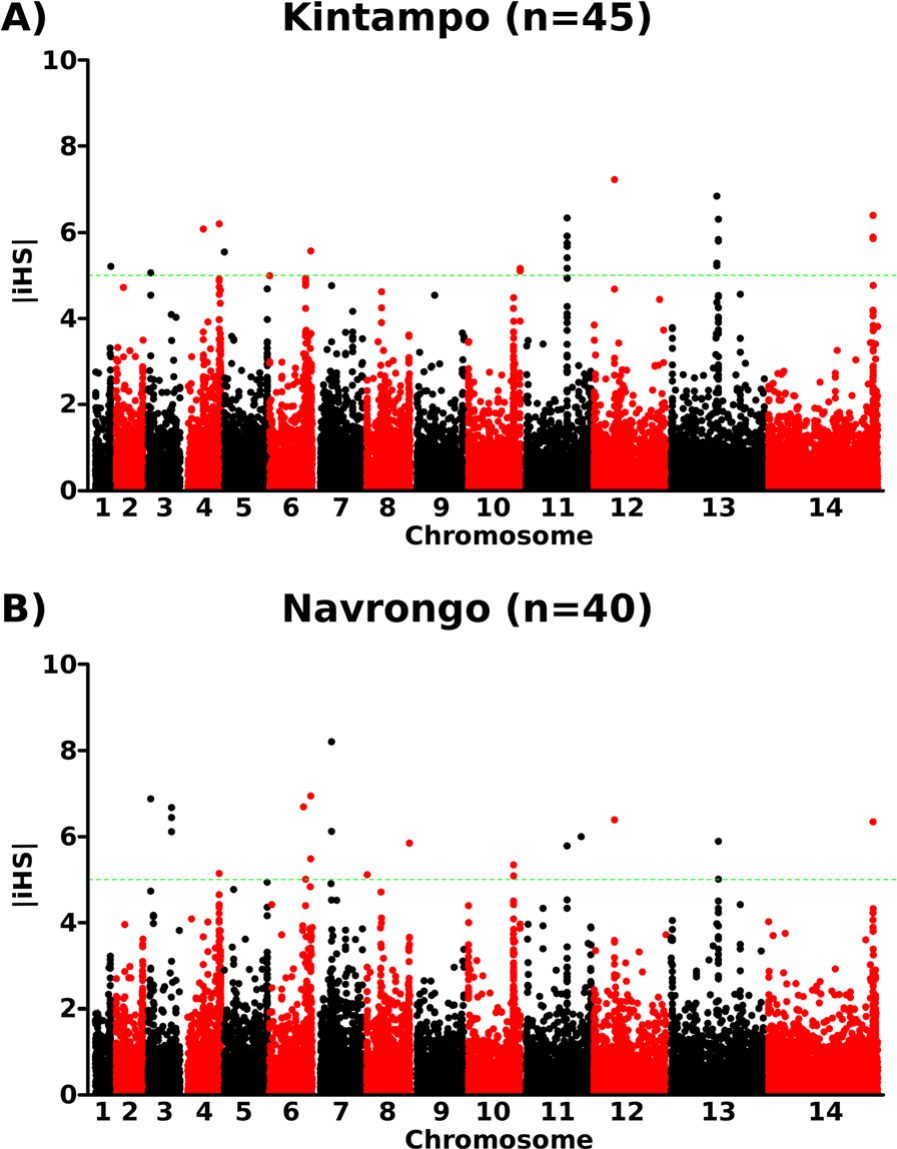
Scan for evidence of recent directional selection by analysis of the standardised integrated haplotype score |iHS| for individual SNPs in Kintampo (*N* = 45) and Navrongo (*N* = 40). Additional file 1: Table S3 gives the co-ordinates and genes in 13 chromosomal regions containing windows of contiguous extended haplotype homozygosity attributed to two or more SNPs within the top 0.1 % of the expected distribution (|iHS| values > 3.29), with at least 1 SNP having |iHS| > 5 in these regions for the combined Ghana population. Most of these chromosomal regions are similar in both populations, as shown schematically in Figure 6

**Fig. 6:**
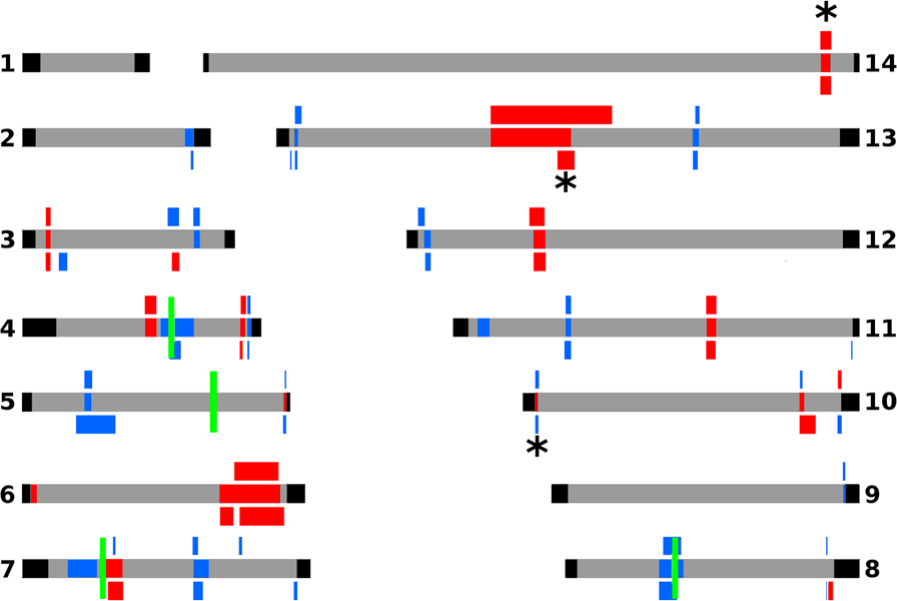
Chromosomal regions with elevated |iHS| values within the combined Ghana population (shaded on chromosome bars), and within each of the local populations (Kintampo shown above and Navrongo below the chromosome bars). Red shading indicates windows containing two or more SNPs within the top 0.1 % of the expected distribution (|iHS| > 3.29) and at least 1 SNP with a |iHS| value > 5. Blue shading indicates windows containing two or more SNPs with |iHS| > 3.29 but none having a value > 5. The regions, and genes within them, are listed in Additional file 1: Table S3. The location of four drug resistance genes mentioned in the text are shown with green markers: *dhfr* (chr 4), *mdr1* (chr 5), *crt* (chr 7) and *dhps* (chr 8). Asterisks show the positions of three elevated |iHS| windows for which the Rsb analysis (Fig. 7) indicated stronger evidence of selection in one or other of the two populations

To identify loci putatively under recent positive selection, indices of long range haplotypes were analysed. Using the standardised integrated haplotype score (|iHS|) for all SNPs above allele frequencies of 5 % in the combined population sample from Ghana (Fig. 5) identified 13 chromosomal regions with two or more SNPs above the top 0.1 % of the randomly expected distribution (|iHS| > 3.29) and at least 1 SNP with |iHS| > 5 (Additional file 1: Table S3). Seven of these regions were also identified in both of the local population samples when applying this cut-off for data from Kintampo and Navrongo separately (Fig. 5 and Additional file 1: Table S3), with a further 4 of the regions observed when a slightly less stringent cut-off was applied (at least 2 SNPs with |iHS| > 3.29) as may be appropriate given the lower sample sizes in the separate populations compared with the combined population. The largest and most strongly supported region of elevated |iHS| values extended over approximately 303 kb on chromosome 6 (Fig. 6 and Additional file 1: Table S3). Another region of high |iHS| values in both population samples covers approximately 50 kb on chromosome 11, having core SNPs within the *ama1* antigen gene. Both of these regions were also detected in analyses of other West African population samples [17, 19, 22]. Elevated |iHS| values were observed around the antifolate drug target genes *dhfr* and *dhps*, as well as the chloroquine resistance transporter gene *crt*, although it was only in the case of *dhps* that a signature covered the gene in both of the local population samples. There was no detectable signature around the multi-drug resistance *mdr1* gene (Fig. 6). A small number of regions had elevated |iHS| values detected in only one or other of the two populations (Fig. 6 and Additional file 1: Table S3), or had an apparently stronger signature in one population than the other, but some of these few apparent differences may be due to random sampling variation.

**Fig. 7:**
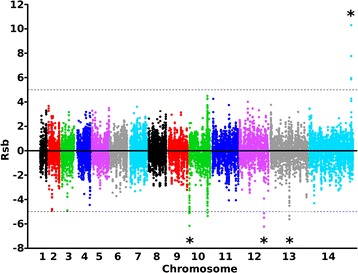
Scan for evidence of population specific directional selection using the Rsb cross-population test for extended haplotypes. Positive values indicate SNPs associated with longer haplotypes in the Kintampo population sample, whereas negative values indicate SNPs associated with longer haplotypes in the Navrongo population sample. One region contains at least 2 SNPs with Rsb values above 5 indicating a potentially stronger signature of selection in Kintampo on chromosome 14 while 3 regions contain at least 2 SNPs with Rsb values below −5 indicating a potentially stronger signature of selection in Navrongo on chromosomes 10, 12 and 13 (marked with asterisks). The regions, and genes within them, are detailed in Fig. 8 and Additional file 1: Table S4. Three of these four regions were associated with high |iHS| windows in both populations (Figs. 5 and 6, Additional file 1: Table S3)

To test for evidence of potential selective differences using an alternative approach, the cross population Rsb metric [29] was applied to compare the average haplotype length associated with each SNP in the two populations. This identified four chromosomal loci with population-specific evidence of directional selection, one in Kintampo and three in Navrongo (Fig. 7 and Additional file 1: Table S4). The Rsb signature for Kintampo within chromosome 14 spans a region of 22 kb covering 5 genes. The Rsb signatures for Navrongo within chromosomes 10, 12 and 13 span 12 kb (four genes), 38 kb (nine genes) and 48 kb (18 genes) respectively.

**Fig. 8:**
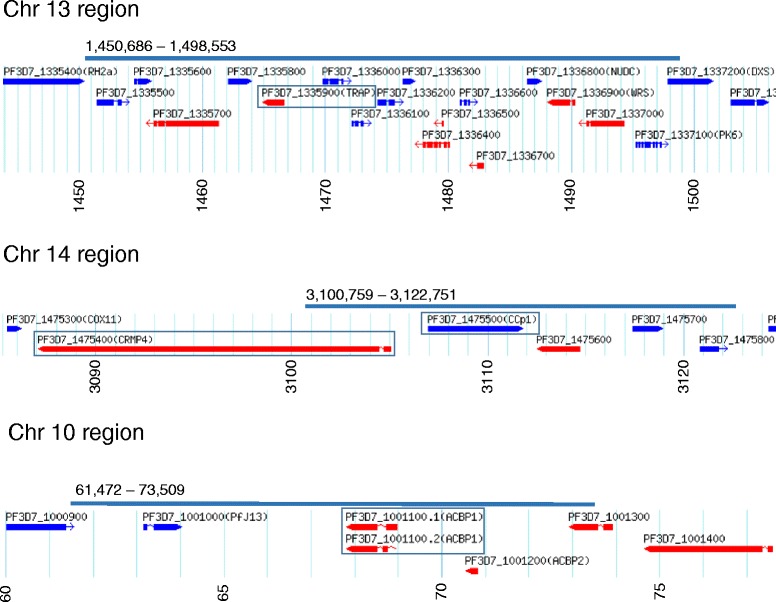
Locations of genes within three narrow genomic regions of *P. falciparum* that had significant Rsb scores indicating differences in selection between Kintampo and Navrongo populations, also coinciding with regions of high |iHS| values in both population samples. Graphics are extracted as snapshots from the respective chromosomal regions in the genome browser at www.plasmodb.org. The chromosomal sequence co-ordinates are shown below each map in kilobase scale. Bars above each map show the extent of the Rsb signatures (specified in Additional file 1: Table S4). Particular genes noted in the Results text are boxed for illustration

Three of the four Rsb signatures (on chromosomes 10, 13 and 14) coincide with regions that had high |iHS| signatures (Figs. 6 and 7). The genes within these regions are shown in Fig. 8 and listed in Additional file 1: Table S4. Further available information on these are accessible using the PlasmoDB browser (www.plasmodb.org) [5]. The functions of most of these genes are unknown, but some have been previously characterised and are potential targets of differential selection (Fig. 8). For example, the region on chromosome 13 contains the *trap* gene (ID Pf3D7_1335900) encoding the thrombospondin related adhesive protein, also known as sporozoite surface protein 2, which is vital for hepatocyte cell invasion and is a leading vaccine candidate antigen to which some naturally acquired immune responses are allele specific [30]. This chromosome 13 region is also immediately adjacent to the *Rh2a*, *Rh2b*, and *msp7* genes encoding polymorphic merozoite proteins involved in erythrocyte invasion. The region on chromosome 14 contains the *crmp4* gene (Pf3D7_1475400) encoding the cysteine repeat modular protein 4 which is essential for mosquito transmission [31], adjacent to the *ccp1* gene (Pf3D7_1475500) encoding an LCCL (*Limulus* coagulation factor C) domain-containing protein expressed in parasite gametocytes [32]. The region on chromosome 10 is in close proximity to the sub-telomere and contains paralogous copies of genes putatively encoding acyl-coA binding proteins (ACBP1 and ACBP2) as well as single members of the *phist* and *hyp* multigene families.

## Discussion

The overall population genetic structure of parasites at both of the endemic sites sampled in Ghana was very similar despite differences in transmission seasonality and local vector species abundances. The within-isolate fixation index *F*_WS_, which is inversely related to the level of genomic complexity per infection, had a similar range of values in each population. The overall levels of within-infection parasite genomic complexity in each of the populations sampled here are similar to those estimated for other highly endemic West African populations in Burkina Faso, Mali, and Guinea, and higher than for a population of lower endemicity in The Gambia [9, 17, 28]. Regarding the individual values, it is worth noting that although an infection with *F*_WS_ value close to 1.0 is dominated by a single population of identical or closely related parasites [33], diverse parasite genotypes may exist at low levels or sequestered in organ capillaries which may become abundant in the peripheral blood after a few hours or days [34].

Similar overall allele frequency distributions observed in both of the sampled populations was not surprising, given their proximity (~350 km apart) and a high level of gene flow generally among West African malaria parasite populations [9, 17, 18]. Genome-wide *F*_ST_ values between the populations were very low, with the small proportion of SNPs having *F*_ST_ values above 0.1 not departing from random expectations accounting for sampling variance. A previous comparison of two West African parasite populations with different levels of endemicity (sites ~1000 km apart in Guinea and The Gambia) also showed very low *F*_ST_ values throughout most of the genome [17]. However, there was exceptional differentiation between the Guinea and Gambia populations over a 15 kb region of chromosome 9 incorporating the gametocyte development 1 gene (*gdv1*, PF3D7_0935400) [17] which is responsible for early initiation of gametocyte development and vital for enabling parasite infection of mosquitoes [35]. There was no differentiation at this locus between the two populations in Ghana studied here (*F*_ST_ = 0.002 for the *gdv1* coding SNP that was previously shown to be differentiated between The Gambia and Guinea).

In contrast, haplotype-based approaches indicated that selection has probably been operating on multiple other loci. Of the 13 genomic windows containing elevated |iHS| scores, marking loci likely to have been under recent directional selection in Ghana, 11 were observed in both of the local population samples. The most significant window identified a large region located towards one end of chromosome 6, as observed previously in other population samples from Senegal, The Gambia, and Guinea [17, 22, 36], although the mechanism and target of selection remains unknown. Another strong signal was due to core SNPs in the *ama1* gene on chromosome 11, encoding an important merozoite target of acquired immunity with extensive sequence polymorphism. This is likely to be subject to occasional positive selection of new alleles added to the repertoire of existing alleles maintained by balancing selection [20]. Strong |iHS| signatures around three major drug resistance genes (*crt*, *dhfr*, and *dhps*) highlight the role of selection by antimalarial treatment across Ghana. Strongest selection on these genes will have occurred when chloroquine and pyrimethamine-sulphadoxine were widely used in antimalarial therapy in Ghana, until Artemisinin Combination Therapy (ACT) replaced them as official first line treatment in 2005, and there may have been local differences in the decay of these signatures following the introduction of ACT. It is also possible that there is some limited ongoing selection, due to use of sulphadoxine – pyrimethamine for intermittent preventive treatment of malaria in pregnancy [37], continued use of chloroquine in some areas despite its proscription [38] or selection by ACT partner drugs which have included amodiaquine and lumefantrine in Ghana.

Three regions, on chromosomes 10, 13 and 14, with high |iHS| values in both of the local populations here were also marked as having stronger evidence of extended haplotypes in one or other of the populations by the Rsb cross-population analysis. This has yielded windows that each contain candidate genes that may be subject to population-specific differences in selection, including the *trap* gene which encodes a vaccine-candidate target of immunity, genes involved in parasite development in mosquitoes, and members of variant-expressed multigene families. Haplotype based tests of directional selection on malaria parasites in West Africa have previously identified selection occurring over very recent time frames, with strong signatures linked to the use of chloroquine and the antifolate drugs sulphadoxine-pyrimethamine as first line malaria therapies [17, 19, 22]. Decay of these signatures following changes in drug use appears to be rapid [22], facilitated by the high recombination rate of *P. falciparum*. Our results here, using a combination of the |iHS| and the Rsb tests, provide evidence of subtle differences in local selection signatures in the two Ghanaian populations that are likely to represent ongoing or very recent selection events. Future studies to investigate selection operating over longer time frames, and heterogeneity among more distantly related populations with potentially differing demography, may employ additional tests including coalescent-based approaches [39–42].

Studies to conduct genome-wide analyses on malaria parasites from a larger number of populations sampled across heterogeneous environments are likely to be able to refine the mapping of loci influenced by local selective processes, and would also indicate the geographical scales of such signatures of selection. Coupling these approaches with epidemiological analyses, including further details of local transmission by different vector species, should facilitate future studies to not only detect signatures of selection but identify the causal processes underlying them.

## Conclusions

This study compares population structure and genomic signatures of selection on malaria parasites in two closely situated but ecologically contrasting endemic areas. Parasite genome sequencing yielded high coverage of more than 120,000 SNPs for population genomic analyses of 45 clinical infection isolates from an area with malaria transmission for most of each year, and 40 isolates from an area with seasonally restricted transmission. The local populations had similar profiles in most respects, particularly with regard to overall SNP allele frequency distributions, and proportions of mixed genotype infections. Eleven different chromosomal regions showed elevated integrated haplotype scores in both populations, but another metric comparing extended haplotype homozygosity (the between-population Rsb index) indicated differences in the strength of the signal between the populations for three of these chromosomal regions. A stronger signal was detected within one population for a narrow region of chromosome 14, whereas a stronger signal was seen in the other population for small regions on chromosomes 10 and 13. These include genes that are potential targets of locally varying recent selection, such as those involved in parasite development in mosquitoes, members of variant-expressed multigene families, and a leading vaccine-candidate target of immunity. Even in a situation of closely situated populations with very similar population structure and shared selection signatures, analysis across heterogeneous environments has potential to refine the mapping of important loci under temporally or spatially varying selection, including those of potential relevance to epidemiology and control of infection.

## Methods

### Sampling of P. falciparum from clinical malaria cases

Blood samples were collected from 146 clinical malaria cases attending Ghana government health facilities in 2011 and 2012, at Kintampo (located 8°3′8″N, 1°44′5″W) in Brong-Ahafo Region of central Ghana, and Navrongo (located 10°53′5″N, 1°5′25″W) in Kassena-Nankana East Municipality, in the Upper East Region of northern Ghana (Fig. 1). Kintampo is located within a holendemic Forest-Savannah transition zone where there is transmission for most of each year, while Navrongo is in a hyperendemic Sudan Savanah region with transmission dependent upon seasonal rainfall. Entomological inoculation rates (EIR) have been estimated to be a few hundred infective bites per person per year in both sites, although this is not a parameter that can be very precisely estimated and it varies from year to year [24, 25].

Approval to collect and analyse the clinical samples was granted by the Ethics committees of the Ghana Health Service, the Noguchi Memorial Institute for Medical Research, University of Ghana, the Kintampo Health Research Centre, the Navrongo Health Research Centre and the London School of Hygiene and Tropical Medicine. Written informed consent was obtained from parents or other legal guardians of all participating children, and additional assent was received from the children themselves if they were 10 years or older. Antimalarial treatment and other supportive care was provided to the children according to the Ghana Health Service guidelines. Patients were eligible for recruitment into the study if they had uncomplicated clinical malaria, were aged 2–14 years, tested positive for *P. falciparum* malaria by Rapid Diagnostic Test (First Response®, Transnational Technologies, UK) or blood smear and had not taken antimalarial drugs during the last 72 h preceding the sample collection. A total of 146 (Kintampo *n* = 88, Navrongo *n* = 58) whole blood samples (up to 5 ml) were collected into heparinised vacutainer tubes (BD Biosciences, CA, USA) and centrifuged. After removal of the plasma and buffy coat, the red cells were depleted of leukocytes by lymphoprep density gradient centrifugation and subsequently passing them through Plasmodipur® filters (EuroProxima, Netherlands), and then frozen at −20 °C. DNA was extracted from each frozen blood sample using the QIAamp blood midi kit (Qiagen, UK) prior to whole genome sequencing.

### Whole genome sequencing of P. falciparum

DNA extracted from the 146 *P. falciparum* positive clinical samples was processed for library preparation and whole genome sequencing at the Wellcome Trust Sanger Institute by paired-end 101 base pair genome sequencing on an Illumina HiSeq platform. Sequence read files for each of these isolates have been deposited in the European Nucleotide Archive (Additional file 1: Table S1). Sequence reads from 101 isolates were of high quality for read-pair mapping to the *P. falciparum* 3D7 reference sequence (v3, October 2012) using BWA-MEM version 0.7.5a [43] with default parameters and SNPs called using SAMTOOLS version 0.1.19 [44] as previously described [17]. For each SNP the majority allele within each infection was identified for use in analysis of population allele frequencies and examination of long range haplotypes. SNPs were excluded from analysis if they were positioned within subtelomeric regions, if they were located within the hypervariable *var*, *rifin* and *stevor* families or if they were positioned within repetitive sequences as identified by Tandem Repeat Finder [45]. The dataset was further filtered to exclude isolates with missing calls at >5 % of all positions and SNPs with calls missing in >10 % of isolates. A total of 85 isolates (Kintampo *n* = 45, Navrongo *n* = 40) and 121712 biallelic SNPs remained for analysis after filtering.

### Statistical analyses

Within-infection genomic diversity in relation to the total local population diversity was determined using the within-isolate *F*_WS_ fixation index [9, 28]. Briefly, for each SNP positioned within a gene, the within isolate expected heterozygosity (Hw) was calculated by determining the total number of reads supporting each allele at that position and comparing these frequencies with the local population heterozygosity (Hs). For this analysis additional filtering of reads was performed as previously described [17]. Isolates with *F*_WS_ scores approaching 1.0 are considered to have a single predominant genotype.

Analysis of allele frequency distributions, including between-population *F*_ST_ [46] and within population Tajima’s D indices [47] was performed using custom R scripts. For *F*_ST_ analysis missing data for some isolates were excluded on a per SNP basis. For Tajima’s D analysis missing data was excluded by removal of individual isolates on a gene by gene basis due to the observation that the majority of missing data clustered within a small number of isolates. Expected genome wide *F*_ST_ distributions were simulated by random assignment of individuals to each population using 1000 replicates and a sampling with replacement strategy. Signatures of directional selection were identified within each population using the standardised |iHS| [48], which was calculated for each SNP with no missing data and a minimum minor allele frequency of 0.05 using the REHH package for the R software environment [49]. Also using data for each SNP with a minimum minor allele frequency of 0.05, a scan for population-specific directional selection was performed using the Rsb metric [29] in the REHH package [49], which assesses the relative haplotype lengths of each SNP between two populations, standardised against the genome wide average. During a previous study comparing two other West African *P. falciparum* populations (in Guinea and The Gambia) [17], we had observed that correction for local recombination rates using LDHat had little effect on the final results, and as this was computationally intensive it was decided that this step was not necessary for the present analysis. Calculation of haplotype breakdown during determination of |iHS| and Rsb scores was terminated if gaps of >20 kb between adjacent SNPs were present within the dataset For both |iHS| and Rsb approaches, putative selection windows were defined by calculating the distance over which the extended haplotypes decayed to a level of 0.05 in each direction [50]. Overlapping windows were combined into continuous windows, while windows supported by only a single high scoring SNP were discarded. For the determination of |iHS| windows, only SNPs with |iHS| > 3.29 were used, with high scoring windows requiring at least 1 SNP with |iHS| > 5. For the analysis of between population differences using the Rsb metric we identified high scoring SNPs as having Rsb > 5 (indicating selection in Kintampo) or Rsb < −5 (indicating selection in Navrongo). We determined EHH decay windows around these SNPs as described above and discarded any windows that included only a single SNP.

### Availability of data and materials

The datasets supporting the results of this article are deposited in the European Nucleotide Archive, with multiple accession numbers provided in the article (listed in Additional file 1: Table S1). The sample identifiers are anonymised and cannot be used to link to any information regarding the identity of the participants in the study.

## Additional files

Additional file 1: Tables S1-S4.
**Table S1. **European Nucleotide Archive accession ID, mean genome wide sequence coverage, and *F*_WS_ scores for each of 146 Ghanaian *P. falciparum* clinical isolates sequenced. Values that could not be determined due to low coverage are shown with a dash (-). **Table S2.** Genome location and allele frequencies for all SNPs with *F*_ST_ > 0.1 between Kintampo and Navrongo. **Table S3.** *P. falciparum* genomic regions with elevated |iHS| values in each of the two local populations and in the combined Ghana dataset. **Table S4.** Windows indicating putative population differences in directional selection as indicated by the rsb metric. Additional file 2: Figures S1-S2.
**Figure S1.** Allele frequency distributions of SNPs within genes in each of the two local *P. falciparum* population samples in Ghana, summarised by the Tajima's D index for each gene with 3 or more SNPs. A. Kintampo population (*N* = 45), with gene indices plotted according to order in the genome. B. Navrongo population (*N* = 40), with indices similarly plotted. **Figure S2.** Principal Component Analysis of genome-wide SNP data (107547 SNPs with no missing data for any of these isolates) shows no difference between *P. falciparum* clinical isolates from the two local populations. Red points show isolates from Kintampo (*N* = 45) and blue points show isolates from Navrongo (*N* = 40). The first three principal components respectively account for 1.90 %, 1.85 %, and 1.83 % of the total observed variation among isolates. Additional file 3:
**Dataset S1.** ***Tajima’s D*** **values in Kintampo and Navrongo populations for all genes with at least 3 SNPs.**
