# Supplementary material for: Comparison of genomic signatures of selection on Plasmodium falciparum between different regions of a country with high malaria endemicity
Source: BMC Genomics. 2015 Jul 16;16(1):527. doi: 10.1186/s12864-015-1746-3 (PMC4502944; doi:10.1186/s12864-015-1746-3)
Supplement: Additional file 1: Tables S1-S4. — Table S1. European Nucleotide Archive accession ID, mean genome wide sequence coverage, and F WS scores for each of 146 Ghanaian P. falciparum clinical isolates sequenced. Values that could not be determined due to low coverage are shown with a dash (-). Table S2. Genome location and allele frequencies for all SNPs with F ST > 0.1 between Kintampo and Navrongo. Table S3. P. falciparum genomic regions with elevated |iHS| values in each of the two local populations and in the combined Ghana dataset. Table S4. Windows indicating putative population differences in directional selection as indicated by the rsb metric. [file 12864_2015_1746_MOESM1_ESM.docx]

Table S1. European Nucleotide Archive accession ID, mean genome wide sequence coverage,

and *F*_WS_ scores for each of 146 Ghanaian *P. falciparum* clinical isolates sequenced. Values that could not be determined due to low coverage are shown with a dash.

The samples and data used in this publication form part of the MalariaGEN Plasmodium falciparum Community Project (<http://www.malariagen.net/projects/parasite/pf>) and the Pf3k project (<http://www.malariagen.net/projects/parasite/pf3k>). Specifically, these data belong to partner study 1083 - Alternative molecular mechanisms for erythrocyte invasion by P. falciparum in Ghana and the contact person is Dr Gordon Awandare. The contributing investigators and data producers have agreed to release the sequence data and country of origin of these samples in the expectation that other researchers will find this useful and will respect the data-sharing principles which are guided by the principles of the Fort Lauderdale Agreement, ([www.genome.gov/Pages/Research/WellcomeReport0303.pdf](http://www.genome.gov/Pages/Research/WellcomeReport0303.pdf)), and are available on the MalariaGEN website (<http://www.malariagen.net/projects/parasite/pf3k/terms-of-use>).

| **Population** | **Sample ID** | **European Nuclotide Archive ID** | **Mean coverage (genome wide)** | ***F*_WS_** |
| --- | --- | --- | --- | --- |
| Kintampo | EIMK077 | ERS188107 | 95 | 0.99 |
| Kintampo | EIMK091 | ERS188114 | 168 | 0.61 |
| Kintampo | EIMK002 | ERS193635 | 52 | - |
| Kintampo | EIMK009 | ERS193640 | 55 | 0.99 |
| Kintampo | EIMK011 | ERS193645 | 115 | 0.99 |
| Kintampo | EIMK012 | ERS193650 | 65 | 0.47 |
| Kintampo | EIMK015 | ERS193655 | 93 | - |
| Kintampo | EIMK017 | ERS193660 | 47 | 0.99 |
| Kintampo | EIMK020 | ERS193665 | 64 | 0.80 |
| Kintampo | EIMK113 | ERS193670 | 109 | 0.99 |
| Kintampo | EIMK081 | ERS193675 | 166 | 0.37 |
| Kintampo | EIMK095 | ERS193680 | 77 | 0.99 |
| Kintampo | EIMK105 | ERS224913 | 188 | 0.48 |
| Kintampo | EIMK119 | ERS246733 | - | - |
| Kintampo | EIMK122 | ERS246734 | - | - |
| Kintampo | EIMK124 | ERS246735 | - | - |
| Kintampo | EIMK128 | ERS246736 | - | - |
| Kintampo | EIMK131 | ERS246737 | 73 | 0.98 |
| Kintampo | EIMK133 | ERS246738 | 153 | 0.99 |
| Kintampo | EIMK134 | ERS246739 | - | - |
| Kintampo | EIMK135 | ERS246740 | 173 | 0.99 |
| Kintampo | EIMK136 | ERS246741 | - | - |
| Kintampo | EIMK139 | ERS246742 | - | - |
| Kintampo | EIMK140 | ERS246743 | - | - |
| Kintampo | EIMK141 | ERS246744 | 179 | 0.44 |
| Kintampo | EIMK144 | ERS246745 | 40 | 0.99 |
| Kintampo | EIMK145 | ERS246746 | - | - |
| Kintampo | EIMK147 | ERS246747 | 193 | 0.99 |
| Kintampo | EIMK149 | ERS246748 | - | - |
| Kintampo | EIMK150 | ERS246749 | 184 | 0.99 |
| Kintampo | EIMK152 | ERS246750 | 42 | - |
| Kintampo | EIMK153 | ERS246751 | 124 | 0.99 |
| Kintampo | EIMK163 | ERS246752 | - | - |
| Kintampo | EIMK168 | ERS246753 | 93 | 0.99 |
| Kintampo | EIMK173 | ERS246754 | 154 | 0.36 |
| Kintampo | EIMK174 | ERS246755 | 99 | 0.99 |
| Kintampo | EIMK176 | ERS246756 | - | - |
| Kintampo | EIMK177 | ERS246757 | 162 | 0.52 |
| Kintampo | EIMK179 | ERS246758 | 200 | 0.99 |
| Kintampo | EIMK200 | ERS246759 | - | - |
| Kintampo | EIMK201 | ERS246760 | 171 | 0.36 |
| Kintampo | EIMK209 | ERS246761 | 106 | 0.55 |
| Kintampo | EIMK210 | ERS246762 | 164 | - |
| Kintampo | EIMK211 | ERS246763 | - | - |
| Kintampo | EIMK212 | ERS246764 | - | - |
| Kintampo | EIMK214 | ERS246765 | 113 | 0.73 |
| Kintampo | EIMK219 | ERS246766 | 156 | 0.96 |
| Kintampo | EIMK220 | ERS246767 | 2 | - |
| Kintampo | EIMK221 | ERS246768 | 193 | 0.49 |
| Kintampo | EIMK227 | ERS246769 | 71 | 0.68 |
| Kintampo | EIMK231 | ERS246770 | 102 | 0.84 |
| Kintampo | EIMK235 | ERS246771 | - | - |
| Kintampo | EIMK239 | ERS246772 | - | - |
| Kintampo | EIMK240 | ERS246773 | 69 | 0.99 |
| Kintampo | EIMK241 | ERS246774 | 146 | 0.99 |
| Kintampo | EIMK242 | ERS246775 | 23 |  |
| Kintampo | EIMK244 | ERS246776 | 122 | 0.99 |
| Kintampo | EIMK247 | ERS246777 | - | - |
| Kintampo | EIMK248 | ERS246778 | - | - |
| Kintampo | EIMK249 | ERS246779 | 145 | 0.99 |
| Kintampo | EIMK251 | ERS246780 | - | - |
| Kintampo | EIMK252 | ERS246781 | - | - |
| Kintampo | EIMK253 | ERS246782 | 122 | 0.54 |
| Kintampo | EIMK255 | ERS246783 | - | - |
| Kintampo | EIMK258 | ERS246784 | 132 | - |
| Kintampo | EIMK263 | ERS246785 | - | - |
| Kintampo | EIMK265 | ERS246786 | - | - |
| Kintampo | EIMK266 | ERS246787 | - | - |
| Kintampo | EIMK267 | ERS246788 | 141 | 0.62 |
| Kintampo | EIMK268 | ERS246789 | - | - |
| Kintampo | EIMK271 | ERS246790 |  | - |
| Kintampo | EIMK274 | ERS246791 | 127 | 0.82 |
| Kintampo | EIMK284 | ERS246792 | 122 | 0.75 |
| Kintampo | EIMK285 | ERS246793 | - | - |
| Kintampo | EIMK286 | ERS246794 | 137 | 0.51 |
| Kintampo | EIMK288 | ERS246795 | - | - |
| Kintampo | EIMK293 | ERS246796 | 175 | 0.98 |
| Kintampo | EIMK296 | ERS246797 | - | - |
| Kintampo | EIMK297 | ERS246798 | - | - |
| Kintampo | EIMK299 | ERS246799 | - | - |
| Kintampo | EIMK300 | ERS246800 | - | - |
| Kintampo | EIMK353 | ERS246801 | - | - |
| Kintampo | EIMK356 | ERS246802 | 52 | 0.99 |
| Kintampo | EIMK359 | ERS246803 | 0 | - |
| Kintampo | EIMK380 | ERS246804 | 172 | 0.92 |
| Kintampo | EIMK384 | ERS246805 | 185 | 0.88 |
| Kintampo | EIMK387 | ERS246806 | - | - |
| Kintampo | EIMK388 | ERS246807 | 204 | 0.99 |
| Navrongo | EIMN001 | ERS311725 | 84 | - |
| Navrongo | EIMN006 | ERS311726 | 141 | 0.99 |
| Navrongo | EIMN008 | ERS311727 | 85 | 0.58 |
| Navrongo | EIMN009 | ERS311728 | 51 | 0.99 |
| Navrongo | EIMN011 | ERS311729 | 110 | 0.26 |
| Navrongo | EIMN013 | ERS311730 | 78 | 0.58 |
| Navrongo | EIMN014 | ERS311731 | 85 | 0.62 |
| Navrongo | EIMN018 | ERS311732 | 79 | 0.99 |
| Navrongo | EIMN020 | ERS311733 | 75 | 0.34 |
| Navrongo | EIMN022 | ERS311734 | 67 | 0.99 |
| Navrongo | EIMN027 | ERS311735 | - | - |
| Navrongo | EIMN031 | ERS311736 | 84 | 0.99 |
| Navrongo | EIMN035 | ERS311737 | 71 | 0.72 |
| Navrongo | EIMN036 | ERS311738 | 71 | 0.99 |
| Navrongo | EIMN037 | ERS311739 | 110 | 0.84 |
| Navrongo | EIMN041 | ERS311740 | 102 | 0.50 |
| Navrongo | EIMN042 | ERS311741 | 62 | 0.22 |
| Navrongo | EIMN043 | ERS311742 | 103 | 0.24 |
| Navrongo | EIMN044 | ERS311743 | 67 | 0.99 |
| Navrongo | EIMN051 | ERS311744 | 69 | 0.99 |
| Navrongo | EIMN054 | ERS311745 | 35 | - |
| Navrongo | EIMN056 | ERS311746 | - | - |
| Navrongo | EIMN059 | ERS311747 | 95 | 0.99 |
| Navrongo | EIMN060 | ERS311748 | 94 | - |
| Navrongo | EIMN062 | ERS311749 | 79 | 0.99 |
| Navrongo | EIMN073 | ERS311750 | 10 | - |
| Navrongo | EIMN079 | ERS311751 | 92 | 0.83 |
| Navrongo | EIMN080 | ERS311752 | 74 | 0.98 |
| Navrongo | EIMN084 | ERS311753 | 62 | 0.52 |
| Navrongo | EIMN086 | ERS311754 | 119 | 0.95 |
| Navrongo | EIMN088 | ERS311755 | 89 | 0.99 |
| Navrongo | EIMN093 | ERS311756 | 74 | 0.80 |
| Navrongo | EIMN094 | ERS311757 | 79 | 0.45 |
| Navrongo | EIMN114 | ERS311758 | 91 | 0.44 |
| Navrongo | EIMN116 | ERS311759 | 141 | 0.92 |
| Navrongo | EIMN120 | ERS311760 | - | - |
| Navrongo | EIMN124 | ERS311761 | 65 | - |
| Navrongo | EIMN131 | ERS311762 | 72 | 0.99 |
| Navrongo | EIMN133 | ERS311763 | - | - |
| Navrongo | EIMN134 | ERS311764 | 108 | 0.99 |
| Navrongo | EIMN137 | ERS311765 | 95 | 0.99 |
| Navrongo | EIMN138 | ERS311766 | 20 | - |
| Navrongo | EIMN151 | ERS311767 | - | - |
| Navrongo | EIMN164 | ERS311768 | 52 | 0.17 |
| Navrongo | EIMN166 | ERS311769 | 64 | 0.60 |
| Navrongo | EIMN167 | ERS311770 | 69 | 0.99 |
| Navrongo | EIMN175 | ERS311771 | - | - |
| Navrongo | EIMN176 | ERS311772 | - | - |
| Navrongo | EIMN180 | ERS311773 | 69 | 0.99 |
| Navrongo | EIMN187 | ERS311774 | - | - |
| Navrongo | EIMN206 | ERS311775 | - | - |
| Navrongo | EIMN216 | ERS311776 | 107 | 0.64 |
| Navrongo | EIMN221 | ERS311777 | - | - |
| Navrongo | EIMN223 | ERS311778 | 59 | 0.27 |
| Navrongo | EIMN226 | ERS311779 | - | - |
| Navrongo | EIMN227 | ERS311780 | - | - |
| Navrongo | EIMN229 | ERS311781 | 87 | 0.88 |
| Navrongo | EIMN231 | ERS311782 | 77 | 0.34 |

Table S2. Genome location and allele frequencies for all SNPs with *F*_ST_ > 0.1 between Kintampo and Navrongo

| **Chromosome** | **Position** | **Gene** | **Reference allele frequency in Kintampo** | **Reference allele frequency in Navrongo** | **F_ST_** | **p value (uncorrected for multiple comparisons)** |
| --- | --- | --- | --- | --- | --- | --- |
| Pf3D7_11_v3 | 1638059 | PF3D7_1140900 | 0.341 | 0.744 | 0.16 | 0.0004 |
| Pf3D7_10_v3 | 1392510 | PF3D7_1035100 | 0.467 | 0.85 | 0.16 | 0.0003 |
| Pf3D7_12_v3 | 1193349 | PF3D7_1229100 | 0.733 | 1 | 0.15 | 0.0003 |
| Pf3D7_06_v3 | 937752 | PF3D7_0623000 | 0.133 | 0.475 | 0.14 | 0.0006 |
| Pf3D7_11_v3 | 241962 | PF3D7_1105600 | 0.622 | 0.25 | 0.14 | 0.0009 |
| Pf3D7_14_v3 | 1666117 | PF3D7_1440800 | 0.467 | 0.125 | 0.14 | 0.0008 |
| Pf3D7_09_v3 | 1113469 | - | 0.089 | 0.4 | 0.13 | 0.0009 |
| Pf3D7_12_v3 | 480382 | - | 1 | 0.775 | 0.13 | 0.0007 |
| Pf3D7_04_v3 | 880115 | PF3D7_0419900 | 0.444 | 0.8 | 0.13 | 0.0009 |
| Pf3D7_12_v3 | 1558740 | - | 0.244 | 0 | 0.13 | 0.0006 |
| Pf3D7_08_v3 | 293019 | - | 0.9778 | 0.73 | 0.13 | 0.0019 |
| Pf3D7_07_v3 | 158694 | PF3D7_0703900 | 0.659 | 0.95 | 0.13 | 0.0009 |
| Pf3D7_06_v3 | 74376 | - | 0.511 | 0.85 | 0.13 | 0.0011 |
| Pf3D7_02_v3 | 382917 | PF3D7_0209100 | 0.9333 | 0.649 | 0.13 | 0.0017 |
| Pf3D7_11_v3 | 1141641 | PF3D7_1129500 | 0.622 | 0.925 | 0.13 | 0.0016 |
| Pf3D7_03_v3 | 514084 | PF3D7_0312100 | 0.711 | 0.975 | 0.13 | 0.0010 |
| Pf3D7_14_v3 | 602114 | PF3D7_1414900 | 0.711 | 0.975 | 0.13 | 0.0010 |
| Pf3D7_07_v3 | 1086194 | PF3D7_0725700 | 0.667 | 0.95 | 0.13 | 0.0011 |
| Pf3D7_10_v3 | 1397893 | - | 0.333 | 0.05 | 0.13 | 0.0011 |
| Pf3D7_02_v3 | 847555 | - | 0.244 | 0.59 | 0.12 | 0.0018 |
| Pf3D7_14_v3 | 60619 | - | 0.9778 | 0.744 | 0.12 | 0.0022 |
| Pf3D7_11_v3 | 1054282 | PF3D7_1126900 | 0.778 | 1 | 0.12 | 0.0013 |
| Pf3D7_11_v3 | 123374 | - | 0.9778 | 0.75 | 0.12 | 0.0041 |
| Pf3D7_14_v3 | 2998127 | - | 0.644 | 0.3 | 0.12 | 0.0022 |
| Pf3D7_13_v3 | 224564 | PF3D7_1304100 | 0.044 | 0.3 | 0.12 | 0.0024 |
| Pf3D7_08_v3 | 1143764 | PF3D7_0826200 | 0.822 | 0.5 | 0.12 | 0.0024 |
| Pf3D7_13_v3 | 885463 | PF3D7_1321300 | 0.533 | 0.85 | 0.12 | 0.0023 |
| Pf3D7_13_v3 | 1629708 | - | 0.9333 | 0.667 | 0.11 | 0.0023 |
| Pf3D7_11_v3 | 1054587 | - | 0.778 | 1 | 0.11 | 0.0017 |
| Pf3D7_14_v3 | 470136 | PF3D7_1411500 | 0.9778 | 0.757 | 0.11 | 0.0043 |
| Pf3D7_14_v3 | 3030631 | PF3D7_1474200 | 0.644 | 0.925 | 0.11 | 0.0034 |
| Pf3D7_14_v3 | 3195310 | PF3D7_1477600 | 0.9318 | 0.667 | 0.11 | 0.0042 |
| Pf3D7_02_v3 | 853523 | - | 0.733 | 0.975 | 0.11 | 0.0020 |
| Pf3D7_08_v3 | 771719 | PF3D7_0816900 | 0.523 | 0.2 | 0.11 | 0.0031 |
| Pf3D7_14_v3 | 1665655 | PF3D7_1440800 | 0.311 | 0.05 | 0.11 | 0.0022 |
| Pf3D7_10_v3 | 1143966 | - | 1 | 0.816 | 0.11 | 0.0030 |
| Pf3D7_05_v3 | 294870 | PF3D7_0507100 | 0.9333 | 0.675 | 0.11 | 0.0043 |
| Pf3D7_12_v3 | 480386 | - | 0.9778 | 0.763 | 0.11 | 0.0045 |
| Pf3D7_01_v3 | 304264 | - | 0.9111 | 0.641 | 0.11 | 0.0033 |
| Pf3D7_10_v3 | 61583 | - | 0.795 | 1 | 0.11 | 0.0028 |
| Pf3D7_04_v3 | 1100954 | PF3D7_0424400 | 0.523 | 0.205 | 0.11 | 0.0034 |
| Pf3D7_04_v3 | 1122147 | PF3D7_0424700 | 0.2 | 0 | 0.11 | 0.0028 |
| Pf3D7_04_v3 | 1137754 | - | 0.8 | 1 | 0.11 | 0.0028 |
| Pf3D7_08_v3 | 1291124 | PF3D7_0830300 | 0.511 | 0.821 | 0.11 | 0.0053 |
| Pf3D7_07_v3 | 1388058 | PF3D7_0732100 | 0.488 | 0.8 | 0.11 | 0.0056 |
| Pf3D7_04_v3 | 1100955 | PF3D7_0424400 | 1 | 0.821 | 0.10 | 0.0037 |
| Pf3D7_06_v3 | 532523 | PF3D7_0612900 | 0.378 | 0.7 | 0.10 | 0.0045 |
| Pf3D7_14_v3 | 2208365 | - | 0.9556 | 0.725 | 0.10 | 0.0051 |
| Pf3D7_05_v3 | 678269 | - | 1 | 0.825 | 0.10 | 0.0038 |
| Pf3D7_09_v3 | 200689 | PF3D7_0904300 | 0 | 0.175 | 0.10 | 0.0038 |
| Pf3D7_14_v3 | 869044 | - | 1 | 0.825 | 0.10 | 0.0038 |

Table S3. *P. falciparum* genomic regions with elevated |iHS| values in each of the two local populations and in the combined Ghana dataset

| **Chromosome** | **Window start** | **Window end** | **Window Size** | **Number of core SNPs within window** | **Number of SNPs with iHS > 5** | **Genes within window** |
| --- | --- | --- | --- | --- | --- | --- |
| **Ghana** |  |  |  |  |  |  |
| Pf3D7_02_v3 | 814702 | 860059 | 45357 | 5 | 0 | PF3D7_0220400 – PF3D7_0221300 |
| Pf3D7_03_v3 | 118650 | 142944 | 24294 | 4 | 2 | PF3D7_0302200 – PF3D7_0302600 |
| Pf3D7_03_v3 | 860997 | 892443 | 31446 | 2 | 0 | PF3D7_0320500 – PF3D7_0321300 |
| Pf3D7_04_v3 | 615405 | 673790 | 58385 | 5 | 1 | PF3D7_0413500 – PF3D7_0415200 |
| Pf3D7_04_v3 | 691961 | 863671 | 171710 | 4 | 0 | PF3D7_0415700 – PF3D7_0419400 |
| Pf3D7_04_v3 | 1093508 | 1118036 | 24528 | 16 | 3 | PF3D7_0424300 – PF3D7_0424600 |
| Pf3D7_04_v3 | 1130287 | 1149024 | 18737 | 13 | 0 | PF3D7_0425000 – PF3D7_0425500 |
| Pf3D7_05_v3 | 311160 | 350957 | 39797 | 2 | 0 | PF3D7_0507600 – PF3D7_0508500 |
| Pf3D7_05_v3 | 1313555 | 1326198 | 12643 | 8 | 1 | PF3D7_0532400 – PF3D7_0532800 |
| Pf3D7_06_v3 | 44518 | 74376 | 29858 | 2 | 1 | PF3D7_0601100 – PF3D7_0601800 |
| Pf3D7_06_v3 | 990033 | 1293881 | 303848 | 33 | 10 | PF3D7_0624400 – PF3D7_0630900 |
| Pf3D7_07_v3 | 227317 | 375091 | 147774 | 3 | 0 | PF3D7_0704600 – PF3D7_0708200 |
| Pf3D7_07_v3 | 406231 | 504867 | 98636 | 6 | 2 | PF3D7_0709000 – PF3D7_0711400 |
| Pf3D7_07_v3 | 860044 | 936965 | 76921 | 3 | 0 | PF3D7_0719700 – PF3D7_0721700 |
| Pf3D7_08_v3 | 468163 | 591335 | 123172 | 7 | 0 | PF3D7_0809200 – PF3D7_0811700 |
| Pf3D7_09_v3 | 1461599 | 1472334 | 10735 | 2 | 0 | PF3D7_0936900 – PF3D7_0937200 |
| Pf3D7_10_v3 | 61472 | 73509 | 12037 | 4 | 1 | PF3D7_1001000 – PF3D7_1001300 |
| Pf3D7_10_v3 | 1387489 | 1413659 | 26170 | 20 | 2 | PF3D7_1035000 – PF3D7_1035700 |
| Pf3D7_11_v3 | 124031 | 185093 | 61062 | 2 | 0 | PF3D7_1102600 – PF3D7_1104200 |
| Pf3D7_11_v3 | 565295 | 593434 | 28139 | 2 | 0 | PF3D7_1114900 – PF3D7_1115700 |
| Pf3D7_11_v3 | 1273301 | 1322708 | 49407 | 12 | 3 | PF3D7_1132900 – PF3D7_1134000 |
| Pf3D7_12_v3 | 88870 | 123645 | 34775 | 3 | 0 | PF3D7_1201200 – PF3D7_1202300 |
| Pf3D7_12_v3 | 638469 | 698683 | 60214 | 4 | 1 | PF3D7_1215700 – PF3D7_1217700 |
| Pf3D7_13_v3 | 93437 | 109859 | 16422 | 7 | 0 | PF3D7_1301700 – PF3D7_1301900 |
| Pf3D7_13_v3 | 1076095 | 1480229 | 404134 | 20 | 6 | PF3D7_1325900 – PF3D7_1336500 |
| Pf3D7_13_v3 | 2089923 | 2124061 | 34138 | 4 | 0 | PF3D7_1352400 – PF3D7_1353000 |
| Pf3D7_14_v3 | 3098922 | 3146976 | 48054 | 13 | 6 | PF3D7_1475400 – PF3D7_1476400 |
|  |  |  |  |  |  |  |
| **Kintampo** |  |  |  |  |  |  |
| Pf3D7_03_v3 | 118942 | 142944 | 24002 | 2 | 1 | PF3D7_0302200 – PF3D7_0302600 |
| Pf3D7_03_v3 | 729186 | 787958 | 58772 | 2 | 0 | PF3D7_0317700 – PF3D7_0318600 |
| Pf3D7_03_v3 | 859215 | 891679 | 32464 | 2 | 0 | PF3D7_0320500 – PF3D7_0321300 |
| Pf3D7_04_v3 | 615405 | 673790 | 58385 | 4 | 1 | PF3D7_0413500 – PF3D7_0415200 |
| Pf3D7_04_v3 | 1096796 | 1123679 | 26883 | 13 | 1 | PF3D7_0424300 – PF3D7_0424700 |
| Pf3D7_04_v3 | 1130187 | 1147311 | 17124 | 10 | 0 | PF3D7_0425000 – PF3D7_0425500 |
| Pf3D7_05_v3 | 311160 | 350957 | 39797 | 2 | 0 | PF3D7_0507600 – PF3D7_0508500 |
| Pf3D7_05_v3 | 1316719 | 1326198 | 9479 | 5 | 0 | PF3D7_0532500 – PF3D7_0532800 |
| Pf3D7_06_v3 | 1064736 | 1286775 | 222039 | 25 | 1 | PF3D7_0626400 – PF3D7_0630700 |
| Pf3D7_07_v3 | 455798 | 469420 | 13622 | 2 | 0 | PF3D7_0710000 – PF3D7_0710200 |
| Pf3D7_07_v3 | 854150 | 882533 | 28383 | 2 | 0 | PF3D7_0719500 – PF3D7_0720300 |
| Pf3D7_07_v3 | 1086811 | 1103957 | 17146 | 6 | 0 | PF3D7_0726000 – PF3D7_0726200 |
| Pf3D7_08_v3 | 488383 | 580045 | 91662 | 3 | 0 | PF3D7_0809600 – PF3D7_0811500 |
| Pf3D7_08_v3 | 1307265 | 1313604 | 6339 | 2 | 0 | PF3D7_0830700 – PF3D7_0830800 |
| Pf3D7_09_v3 | 1460448 | 1472334 | 11886 | 2 | 0 | PF3D7_0936900 – PF3D7_0937200 |
| Pf3D7_10_v3 | 61472 | 82375 | 20903 | 2 | 0 | PF3D7_1001000 – PF3D7_1001500 |
| Pf3D7_10_v3 | 1389808 | 1405094 | 15286 | 9 | 0 | PF3D7_1035100 – PF3D7_1035400 |
| Pf3D7_10_v3 | 1581310 | 1599885 | 18575 | 3 | 2 | PF3D7_1039300 – PF3D7_1039900 |
| Pf3D7_11_v3 | 565295 | 592345 | 27050 | 2 | 0 | PF3D7_1114900 – PF3D7_1115700 |
| Pf3D7_11_v3 | 1271031 | 1322708 | 51677 | 15 | 6 | PF3D7_1132800 – PF3D7_1134000 |
| Pf3D7_12_v3 | 56815 | 91607 | 34792 | 2 | 0 | PF3D7_1200700 – PF3D7_1201200 |
| Pf3D7_12_v3 | 617072 | 693042 | 75970 | 2 | 1 | PF3D7_1214800 – PF3D7_1217400 |
| Pf3D7_13_v3 | 93437 | 127110 | 33673 | 4 | 0 | PF3D7_1301700 – PF3D7_1302300 |
| Pf3D7_13_v3 | 1076095 | 1685846 | 609751 | 17 | 6 | PF3D7_1325900 – PF3D7_1342900 |
| Pf3D7_13_v3 | 2102174 | 2124673 | 22499 | 3 | 0 | PF3D7_1352700 – PF3D7_1353100 |
| Pf3D7_14_v3 | 3097463 | 3152804 | 55341 | 11 | 3 | PF3D7_1475400 – PF3D7_1476600 |
|  |  |  |  |  |  |  |
| **Navrongo** |  |  |  |  |  |  |
| Pf3D7_02_v3 | 845983 | 860144 | 14161 | 3 | 0 | PF3D7_0220900 – PF3D7_0221300 |
| Pf3D7_03_v3 | 118067 | 142239 | 24172 | 2 | 1 | PF3D7_0302100 – PF3D7_0302600 |
| Pf3D7_03_v3 | 183105 | 226751 | 43646 | 3 | 0 | PF3D7_0303500 – PF3D7_0304700 |
| Pf3D7_03_v3 | 751533 | 788886 | 37353 | 3 | 3 | PF3D7_0318200 PF3D7_0318700 |
| Pf3D7_04_v3 | 738775 | 796268 | 57493 | 2 | 0 | PF3D7_0416900 – PF3D7_0418000 |
| Pf3D7_04_v3 | 1092186 | 1106637 | 14451 | 13 | 1 | PF3D7_0424300 – PF3D7_0424400 |
| Pf3D7_04_v3 | 1130457 | 1140274 | 9817 | 7 | 0 | PF3D7_0425000 – PF3D7_0425200 |
| Pf3D7_05_v3 | 269798 | 468880 | 199082 | 2 | 0 | PF3D7_0506500 – PF3D7_0511000 |
| Pf3D7_05_v3 | 1309681 | 1326198 | 16517 | 4 | 0 | PF3D7_0532300 – PF3D7_0532800 |
| Pf3D7_06_v3 | 993028 | 1060463 | 67435 | 3 | 1 | PF3D7_0624500 – PF3D7_0626200 |
| Pf3D7_06_v3 | 1091436 | 1314830 | 223394 | 20 | 3 | PF3D7_0627100 – PF3D7_0631100 |
| Pf3D7_07_v3 | 430421 | 507284 | 76863 | 7 | 2 | PF3D7_0709600 – PF3D7_0711500 |
| Pf3D7_07_v3 | 857507 | 908782 | 51275 | 4 | 0 | PF3D7_0719600 – PF3D7_0721000 |
| Pf3D7_07_v3 | 1362925 | 1380895 | 17970 | 2 | 0 | PF3D7_0731500 – PF3D7_0731800 |
| Pf3D7_08_v3 | 468163 | 559536 | 91373 | 6 | 0 | PF3D7_0809200 – PF3D7_0811100 |
| Pf3D7_08_v3 | 1308068 | 1311760 | 3692 | 2 | 0 | PF3D7_0830800 |
| Pf3D7_08_v3 | 1318302 | 1342209 | 23907 | 4 | 1 | PF3D7_0830900 – PF3D7_0831300 |
| Pf3D7_10_v3 | 61371 | 79028 | 17657 | 4 | 0 | PF3D7_1001000 – PF3D7_1001400 |
| Pf3D7_10_v3 | 1389367 | 1470239 | 80872 | 16 | 2 | PF3D7_1035000 – PF3D7_1037100 |
| Pf3D7_10_v3 | 1579304 | 1599885 | 20581 | 2 | 0 | PF3D7_1039300 – PF3D7_1039900 |
| Pf3D7_11_v3 | 559110 | 593444 | 34334 | 4 | 0 | PF3D7_1114800 – PF3D7_1115700 |
| Pf3D7_11_v3 | 1271031 | 1318566 | 47535 | 4 | 1 | PF3D7_1132800 – PF3D7_1133900 |
| Pf3D7_11_v3 | 2001090 | 2004010 | 2920 | 2 | 0 | PF3D7_1149600 |
| Pf3D7_12_v3 | 92538 | 123645 | 31107 | 2 | 0 | PF3D7_1201300 – PF3D7_1202300 |
| Pf3D7_12_v3 | 638469 | 698639 | 60170 | 3 | 1 | PF3D7_1215700 – PF3D7_1217700 |
| Pf3D7_13_v3 | 69208 | 78593 | 9385 | 2 | 0 | PF3D7_1301100 – PF3D7_1301200 |
| Pf3D7_13_v3 | 92800 | 106114 | 13314 | 5 | 0 | PF3D7_1301600 – PF3D7_1301800 |
| Pf3D7_13_v3 | 1411850 | 1497244 | 85394 | 11 | 2 | PF3D7_1334800 – PF3D7_1337100 |
| Pf3D7_13_v3 | 2089923 | 2116599 | 26676 | 3 | 0 | PF3D7_1352400 – PF3D7_1352900 |
| Pf3D7_14_v3 | 3097463 | 3151888 | 54425 | 8 | 1 | PF3D7_1475400 – PF3D7_1476500 |

Table S4. Windows indicating putative population differences in directional selection as indicated by the rsb metric

| **Population** | **Chromosome** | **Window Start** | **Window End** | **Window Size (bp)** | **Number of core SNPs** | **Genes covered** |
| --- | --- | --- | --- | --- | --- | --- |
| Kintampo | Pf3D7_14_v3 | 3100739 | 3122751 | 22012 | 4 | PF3D7_1475400 – PF3D7_1475800 |
| Navrongo | Pf3D7_10_v3 | 61472 | 73509 | 12037 | 4 | PF3D7_1001000 – PF3D7_1001300 |
| Navrongo | Pf3D7_12_v3 | 1828127 | 1865629 | 37502 | 4 | PF3D7_1243700 – PF3D7_1244500 |
| Navrongo | Pf3D7_13_v3 | 1450686 | 1498553 | 47867 | 2 | PF3D7_1335500 – PF3D7_1337200 |
