# Supplementary material for: Comparison of genomic signatures of selection on Plasmodium falciparum between different regions of a country with high malaria endemicity
Source: BMC Genomics. 2015 Jul 16;16(1):527. doi: 10.1186/s12864-015-1746-3 (PMC4502944; doi:10.1186/s12864-015-1746-3)

Supplementary Figures

Supplementary Figure S1. Allele frequency distributions of SNPs within genes in each of the two local *P. falciparum* population samples in Ghana. Kintampo population (N = 45), with gene indices plotted according to order in the genome. B. Navrongo population (N = 40), with indices similarly plotted.

Supplementary Figure S2. Principal Component Analysis of genome-wide SNP data (107547 SNPs with no missing data for any of these isolates) shows no difference between *P. falciparum* clinical isolates from the two local populations. Red points show isolates from Kintampo (N = 45) and blue points show isolates from Navrongo (N = 40). The first three principal components respectively account for 1.90%, 1.85%, and 1.83% of the total observed variation among isolates.

Supplementary Figure S1

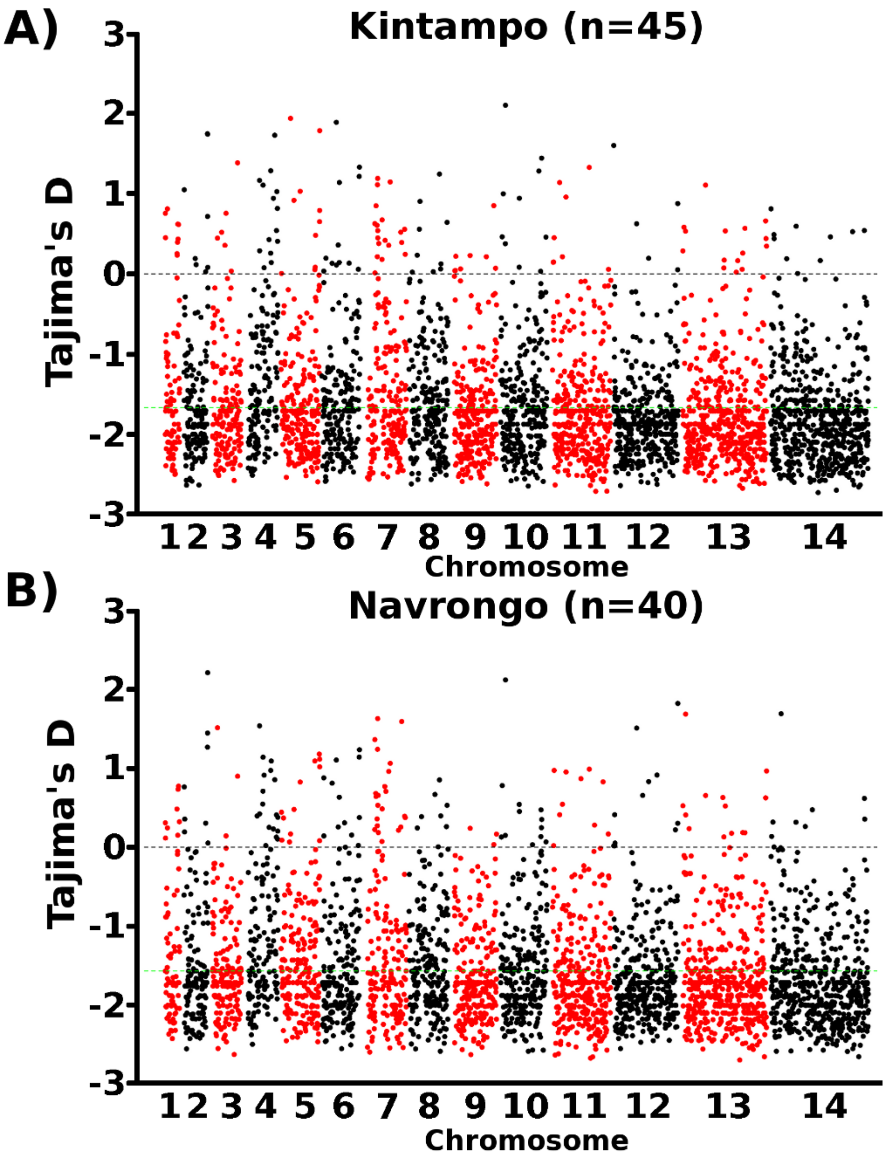

Supplementary Figure S2

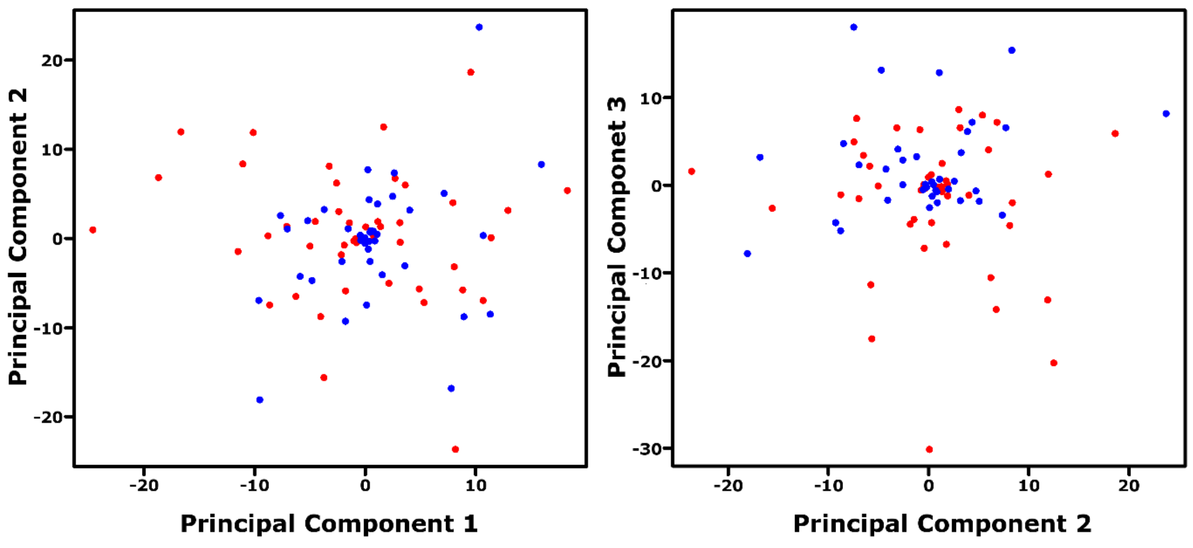

Supplement: Additional file 2: Figures S1-S2. — Figure S1. Allele frequency distributions of SNPs within genes in each of the two local P. falciparum population samples in Ghana, summarised by the Tajima's D index for each gene with 3 or more SNPs. A. Kintampo population (N = 45), with gene indices plotted according to order in the genome. B. Navrongo population (N = 40), with indices similarly plotted. Figure S2. Principal Component Analysis of genome-wide SNP data (107547 SNPs with no missing data for any of these isolates) shows no difference between P. falciparum clinical isolates from the two local populations. Red points show isolates from Kintampo (N = 45) and blue points show isolates from Navrongo (N = 40). The first three principal components respectively account for 1.90 %, 1.85 %, and 1.83 % of the total observed variation among isolates. [file 12864_2015_1746_MOESM2_ESM.pdf]
